# Platelet transfusions in cancer patients with hypoproliferative thrombocytopenia in the intensive care unit

**DOI:** 10.1186/s13613-015-0088-2

**Published:** 2015-12-01

**Authors:** Bassem Habr, Julien Charpentier, Benoît Champigneulle, Agnès Dechartres, Fabrice Daviaud, Guillaume Geri, Alain Cariou, Jean-Daniel Chiche, Jean-Paul Mira, Frédéric Pène

**Affiliations:** Réanimation Médicale, Hôpital Cochin, Assistance Publique-Hôpitaux de Paris, 27 rue du Faubourg Saint-Jacques, 75014 Paris, France; Faculté de Médecine, Université Paris Descartes, Sorbonne Paris Cité, Paris, France; Centre d’épidémiologie et de recherche clinique, Hôtel-Dieu, Assistance Publique-Hôpitaux de Paris, Paris, France; Institut Cochin, INSERM U1016, CNRS UMR8104, Paris, France

**Keywords:** Thrombocytopenia, Intensive care unit, Cancer, Bleeding, Transfusion

## Abstract

**Background:**

Thrombocytopenia is a frequent finding in critically ill cancer patients for whom indications of platelet transfusions are unclear. We herein addressed the current practices in platelet transfusion and the risk of bleeding in cancer patients with hypoproliferative thrombocytopenia in the intensive care unit (ICU).

**Methods:**

A retrospective monocenter study over a 7-year period was conducted in a medical ICU. Adult patients with malignancies and hypoproliferative thrombocytopenia, and who received at least one platelet concentrate during their ICU stay, were included.

**Results:**

296 patients were included and received a total of 904 platelet transfusions, for prophylactic indications in 300 (33.2 %) episodes, for securing an invasive procedure in 257 (28.4 %), and for treatment of minor to major bleeding manifestations in 347 (38.4 %). Most prophylactic transfusions (80 %) were performed at platelet count thresholds below 10–20 × 10^9^/L. Platelet increments were generally low in all three indications, 10 (interquartile range 2–25), 11 (2–25), and 8 (0–21) × 10^9^/L, respectively. A total of 97 major ICU-acquired bleeding events occurred in 40 patients. About half of those bleeding episodes (54.7 %) occurred at platelet counts below 20 × 10^9^/L. However, neither low admission platelet count nor low nadir platelet counts were predictive of ICU-acquired bleeding. The in-ICU mortality rate tended to be higher in patients with severe ICU-acquired bleeding events (50 vs. 36 %).

**Conclusions:**

Most prophylactic platelet transfusions were given using thresholds of 10–20 × 10^9^/L in critically ill thrombocytopenic cancer patients. The individual risk of ICU-acquired severe bleeding appears hardly predictable with the depth of thrombocytopenia.

## Background

Thrombocytopenia is a frequent finding in critically ill patients, either present at the time of intensive care unit (ICU) admission or further acquired during the ICU stay [1]. Thrombocytopenia carries major implications in terms of diagnostic investigations, prognosis, and specific treatment with particular emphasis on platelet transfusions. The mechanisms leading to thrombocytopenia in the critically ill are mostly peripheral through consumption or destruction [2]. The poor prognostic value of thrombocytopenia has been largely emphasized in the ICU. Most interestingly, it has been shown that relative thrombocytopenia, as defined by a 30 % drop in platelet count regardless of baseline and final values, shares similar pathophysiological and prognostic significance with absolute thrombocytopenia [3, 4]. This suggests that the process leading to platelet consumption is more important than the platelet count by itself. With respect to the risk of bleeding, it is striking that the risk of bleeding in ICU patients is poorly related to the depth of thrombocytopenia [2, 4, 5]. However, such studies were performed in cohorts of critically ill patients in whom peripheral mechanisms leading to thrombocytopenia were largely predominant [2, 5]. In contrast, a number of ICU critically ill patients primarily exhibit decreased platelet production as a consequence of underlying malignant diseases and their myelosuppressive treatments. The relation between platelet count and bleeding in this setting has not been addressed.

The only goal of platelet transfusions is to prevent or to treat bleeding. To this day, indications of platelet transfusions in the ICU remain highly speculative. Recently, the French Intensive Care Society provided some expert recommendations guiding platelet transfusions in the ICU [6]. The platelet count threshold for securing invasive procedures or treating severe bleeding was set at 50 × 10^9^/L. As for prophylactic platelet transfusions, the guidelines did not retain any indication in patients with peripheral thrombocytopenia and proposed a platelet count threshold of 20 × 10^9^/L for hypoproliferative thrombocytopenia. Of note, such a restrictive transfusion policy was only based on studies performed in hematologic patients in stable conditions and may not apply to critically ill patients. We herein aimed to address the current practices in platelet transfusion and the risk of bleeding in cancer patients with hypoproliferative thrombocytopenia in a medical intensive care unit.

## Patients and methods

### Patients and setting

This retrospective observational study was carried out over a 7-year period (December 2006 to February 2014) in a 24-bed tertiary medical ICU. The unit is located in a university hospital that comprises hematology and oncology departments, and has an average of 1600 admissions per year, with a large majority (>85 %) of medical patients. Adult patients with hematologic malignancies or solid tumors with presumed or proved central thrombocytopenia as defined by a platelet count below 150 × 10^9^/L, and who received at least one platelet concentrate during the ICU stay, were included. Patients with thrombocytopenia from exclusive peripheral origin were not included. Senior staffing of the unit remained unchanged during the study period. The study was conducted in compliance with the Declaration of Helsinki principles and with the French regulation of clinical research. The ethics committee of the French Intensive Care Society (Société de Réanimation de Langue Française, CE SRLF #15-17) approved the protocol and waived the need for patients’ consent because of the retrospective observational design of the study.

### Collection of data

Data were computed from medical files and extracted from the patient data management system (CliniSoft, GE Healthcare). For each patient, the following baseline data were collected: demographics (age and gender), type of malignancy (solid tumor or hematologic malignancy), and the presence of other comorbidities such as chronic organ dysfunctions. The presumed or proved mechanisms of central thrombocytopenia were also collected.

The characteristics related to the ICU stay were collected and included the main reasons for ICU admission, the main biological values on admission such as platelet count and leukopenia that was defined by leukocyte count <1000/mm^3^ and the requirements for organ failure supports during the stay in the ICU. Severity at admission was assessed by Simplified Acute Physiology Score II (SAPS II) and Sequential Organ Failure Assessment (SOFA) score during the first 24 h following ICU admission [7, 8]. We also calculated a modified SOFA score without the platelet component.

### Definitions of bleeding events

Bleeding events were recorded and graded from 0 to 4 according to the World Health Organization (WHO) classification [6, 9]. Briefly, grades 0 and 1 meant no or minor bleeding, respectively. Grade 2 accounted for mild bleeding yet requiring medical intervention but no transfusion of red blood cell (RBC) concentrates. Grade 3 accounted for gross blood loss responsible for advent or deterioration of organ failures and/or requiring transfusion of up to two RBC concentrates within 24 h of onset. Grade 4 accounted for debilitating blood loss including central nervous system or retinal hemorrhages, massive bleeding requiring transfusion of more than two RBC concentrates within 24 h of onset, and more generally fatal bleeding from any source. Major bleeding events were defined as WHO grade 3 and 4.

### Intended management of platelet transfusions

Platelet transfusions were performed using ABO-compatible and leukodepleted concentrates, prepared either from pooled whole-blood donations or from single donor apheresis. The respective indications of pooled and apheresis concentrates depended on the clinical situation, an eventual anti-HLA immunization or the availability of blood products. Platelet dose per transfusion was calculated according to body weight (0.5–0.7 × 10^11^ platelets per 7 kg body weight) [6, 10].

Indications of platelet transfusions were distributed into the three following categories: prophylactic, securing an invasive procedure, and therapeutic. Prophylactic transfusions aimed at maintaining platelet count above 10–20 × 10^9^/L. Transfusions to secure invasive procedures were performed before, during, or after the intervention, without systematic subsequent checking for an effective increment in platelet count. Therapeutic platelet transfusion was performed in case of active bleeding. For each platelet transfusion, pre- and post-transfusion platelet counts were computed from the last value prior to the transfusion and from the subsequent morning value, respectively. Platelet increment was estimated by calculating the absolute difference between post- and pre-transfusion platelet counts. We investigated the serious adverse events attributable to platelet transfusion as mentioned in the medical files and/or through reporting to our hospital’s hemovigilance.

### Statistical analysis

Continuous and categorical variables were expressed as median and interquartile range or as number and percentage, respectively. Comparisons were performed by using non-parametric (Mann–Whitney) and *χ*^2^ tests as appropriate.

## Results

### Study population

During the 7-year study period, 684 patients from our ICU received at least one platelet transfusion. Among them, 296 patients had hypoproliferative thrombocytopenia in the setting of malignancy and received a total of 904 platelet transfusions. Their baseline characteristics are shown in Table 1. Most patients (81.7 %) had underlying hematologic malignancies, of whom 42 (17.3 %) were hematopoietic stem cell transplantation recipients. The median platelet count at the time of ICU admission was 29 (15–54) × 10^9^/L and the nadir platelet count during the ICU stay was 12 (6–23) × 10^9^/L. According to the clinical background of patients, central thrombocytopenia was presumably related to myelosuppressive chemotherapy and/or radiotherapy in most cases. In addition, bone marrow explorations resulted in identification of alternative mechanisms of bone marrow suppression in 73 (24.5 %) patients. As expected, a number of patients also displayed leukopenia. The main cause of admission to the ICU was sepsis in 139 (47 %) patients, while 17 (5.7 %) were admitted for major hemorrhage (Table 2). Patients generally presented with severe conditions as shown by admission severity scores as well as frequent requirement of organ failure supports during the ICU stay. The in-ICU mortality rate was 37.8 %.

**Table 1:** Baseline characteristics of patients

Variables	296 patients
Age (years)	62 (53–70)
Male gender	196 (66 %)
Underlying malignancies	
Hematologic malignancies	242 (81.7 %)
Acute leukemia	99 (33.4 %)
Lymphoma	96 (32.4 %)
Multiple myeloma	28 (9.4 %)
Miscellaneous	19 (6.4 %)
Solid tumors	54 (18.3 %)
Uro-genital	17 (5.7 %)
Digestive	15 (5 %)
Pulmonary	9 (3 %)
Breast	6 (2 %)
Bone and cartilage	4 (1.3 %)
Head and neck	4 (1.3 %)
Hematopoietic stem cell transplantation	42 (14.2 %)
Other comorbidities	
Chronic heart failure	61 (20.6 %)
Chronic liver disease	13 (4.4 %)
Chronic and end-stage renal failure	13 (4.4 %)
Human immunodeficiency virus infection	6 (2 %)
Solid organ transplantation	1 (0.3 %)
Anticoagulant and antiplatelet medications	69 (23.3 %)
Prophylactic anticoagulant	21 (7.1 %)
Curative anticoagulant	27 (9.1 %)
Antiplatelet	21 (7.1 %)
Mechanisms of central thrombocytopenia	
Chemo- or radiotherapy-induced	223 (75.3 %)
Bone marrow infiltration by malignant cells	55 (18.5 %)
Hemophagocytosis	10 (3.4 %)
Drug-induced (allergic)	4 (1.3 %)
Myelofibrosis	4 (1.3 %)

Variables are expressed as median (interquartile range) or number (percentage)

**Table 2:** Characteristics of ICU admission and management of organ failures

Variables	296 patients
Reasons for ICU admission	
Sepsis	139 (47 %)
Acute respiratory failure	67 (22.6 %)
Severe hemorrhage	17 (5.7 %)
Acute renal failure	16 (5.4 %)
Others	57 (19.2 %)
Admission severity scores	
SAPS II (points)	51 (36–69)
SOFA (points)	7 (5–10)
Modified SOFA (points)	4 (2–7)
Admission biological values	
Platelet count (×10^9^/L)	29 (15–54)
Leukopenia	117 (39.5 %)
Prothrombin time (%)	65 (51–77)
Serum urea (mmol/L)	10 (6.8–17.8)
Serum creatinine (μmol/L)	108 (72–184)
Serum bilirubin (μmol/L)	19 (11–42)
Organ failure supports during ICU stay	
Non-invasive ventilation	120 (40.5 %)
Invasive mechanical ventilation	181 (61.1 %)
Vasopressors	187 (63.2 %)
Renal replacement therapy	120 (40.5 %)
Platelet transfusion management	
Nadir platelet count during ICU stay (10^9^/L)	12 (6–23)
Total number of platelet transfusions	2 (1–4)
In-ICU length of stay (days)	5 (2.3–13.2)
In-ICU mortality	112 (37.8 %)

Variables are expressed as median (interquartile range) or number (percentage). The modified SOFA score did not include the platelet component

*ICU* intensive care unit, *SAPS II* simplified acute physiology score II, *SOFA* sequential organ failure assessment score

### Indications for platelet transfusions

**Fig. 1:**
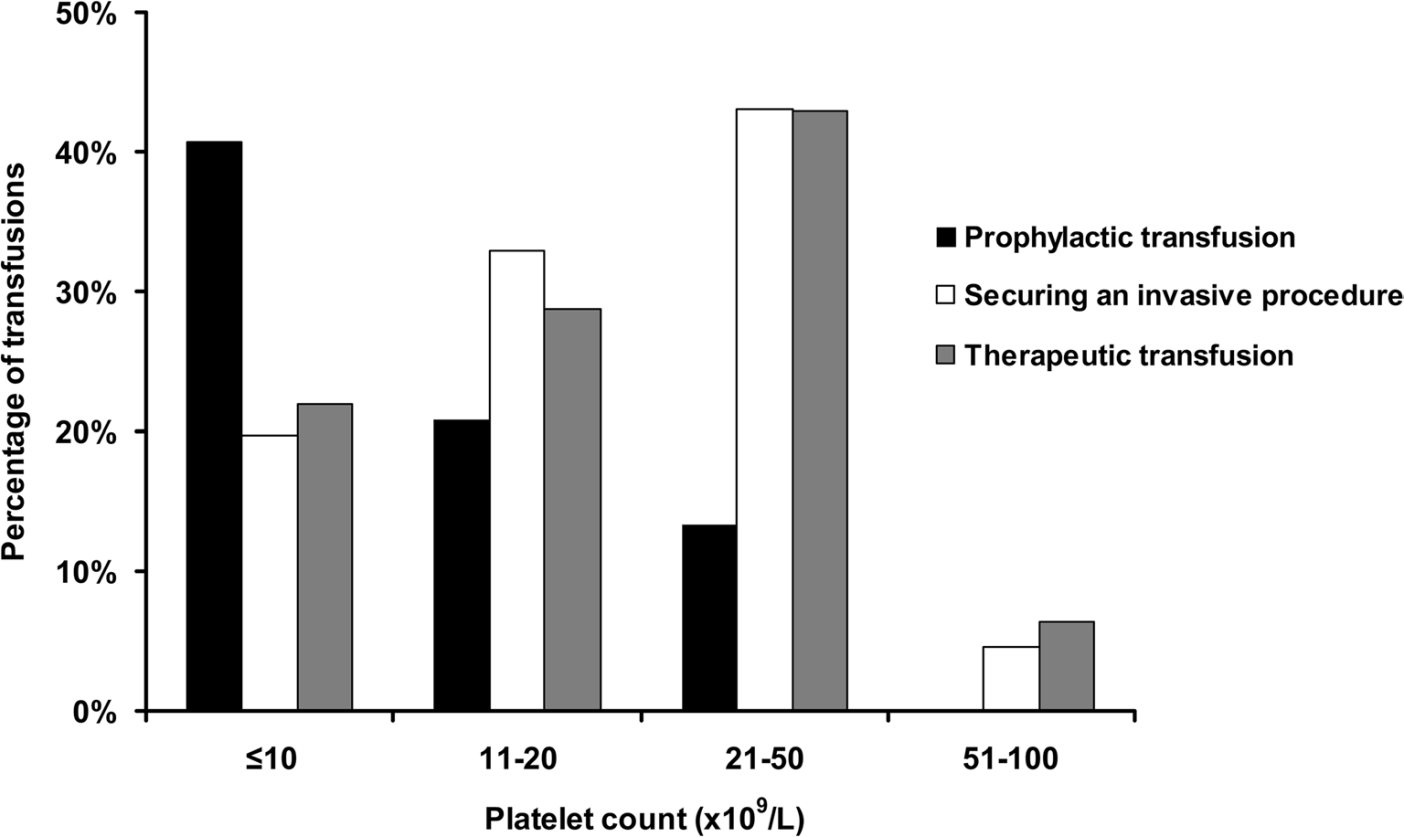
Distribution of pre-transfusion platelet counts for the three indications of transfusion: prophylactic (*n* = 300), securing an invasive procedure (*n* = 257) and therapeutic (*n* = 347)

**Fig. 2:**
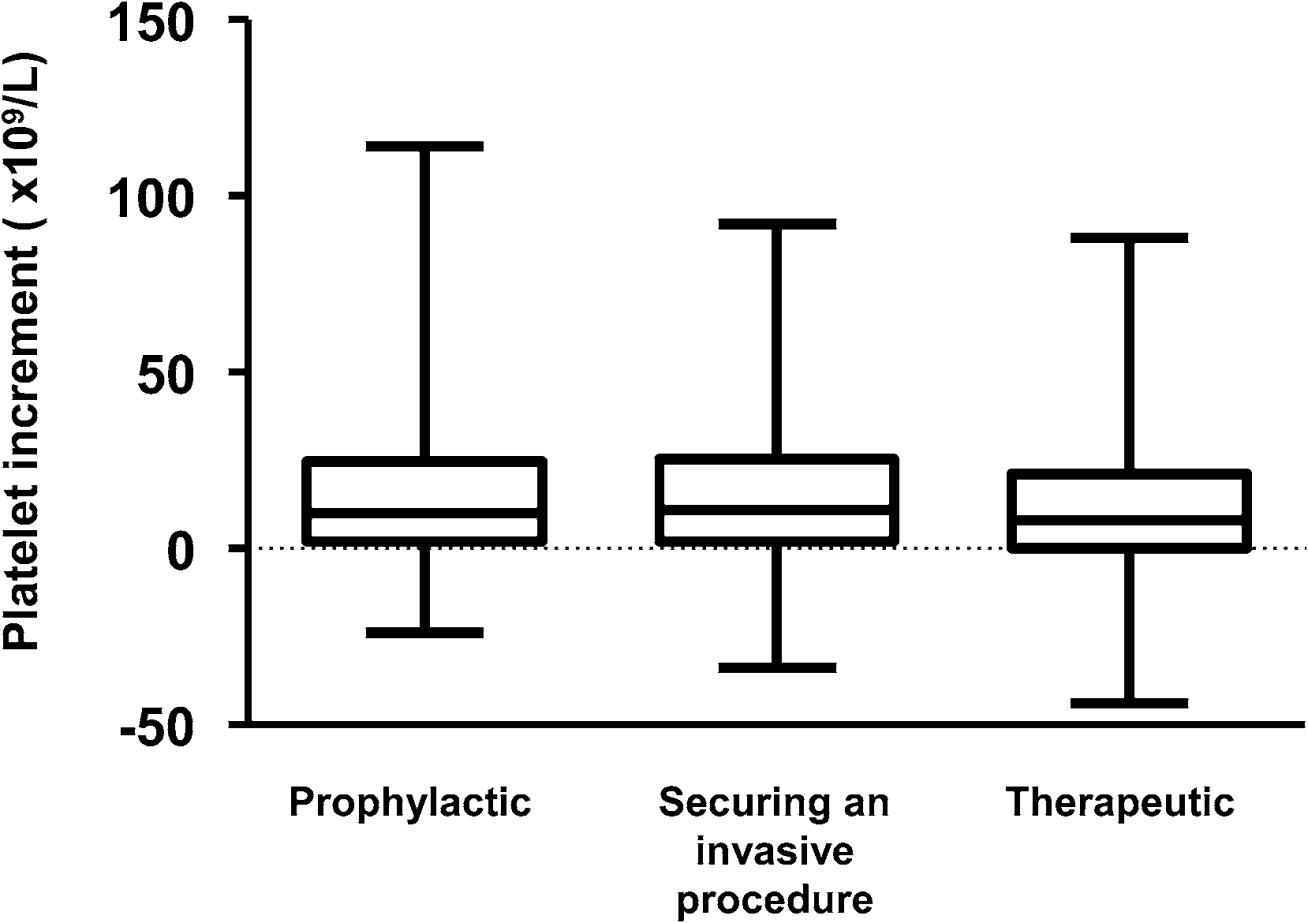
Platelet count recovery according to indications for platelet transfusions

Indications for the 904 platelet transfusions were distributed into prophylactic in 300 (33.2 %) episodes, securing an invasive procedure in 257 (28.4 %) episodes, mainly for catheter insertion (42.3 %) and surgery (27 %), and therapeutic for minor to major bleeding manifestations in 347 (38.4 %) episodes. Grade 1, 2, 3, and 4 bleeding events accounted for 12, 135, 132, and 68 episodes of therapeutic platelet transfusions, respectively. Figure 1 represents the distribution of platelet transfusions according to pre-transfusion platelet count in those three indications. Most prophylactic transfusions (80 %) were performed when platelet counts were below 20 × 10^9^/L. Figure 2 displays platelet recovery as estimated by pre- and post-transfusion platelet counts and the resulting platelet count increment in the three indications of platelet transfusions. Platelet transfusions were generally well tolerated as only five serious adverse events were reported, all being immediate respiratory deterioration presumably related to volume overload.

### ICU-acquired major bleeding events

Seventeen patients who had been primarily admitted for severe hemorrhage were excluded from the following analysis regarding ICU-acquired bleeding. Among the remaining 279 patients, a total of 97 WHO grade 3 and 4 bleeding events occurred in 40 patients (Table 3). About half of severe bleeding episodes (54.7 %) occurred in the setting of profound thrombocytopenia with platelet counts below 20 × 10^9^/L. Concurrent therapeutic and prophylactic anticoagulant or antiplatelet agents were likely to have contributed to bleeding in only 8.2 % of episodes. In an attempt to identify some determinants of ICU-acquired severe bleeding, we compared the main characteristics of patients who did and did not develop severe bleeding during the ICU stay (Table 4). Patients who developed severe bleeding were more likely to be leukopenic, and both admission and nadir platelet counts were higher in patients who bled. ICU-acquired severe bleeding tended to be associated with increased in-ICU mortality (50 vs. 36 %).

**Table 3:** Characteristics of the 97 ICU-acquired severe bleeding events in 40 patients

Variable	ICU-acquired severe bleeding events (*n* = 97)
WHO bleeding classification	
Grade 3	64 (66 %)
Grade 4	33 (34 %)
Timing of bleeding	
Time between admission and bleeding (days)	4 (1–9.5)
Sites of bleeding	
Digestive	32 (33 %)
Pulmonary	22 (22.7 %)
Site of invasive procedure	13 (13.4 %)
Central nervous system	5 (5.1 %)
Urinary	8 (8.2 %)
Others	17 (17.5 %)
Platelet count at the time of bleeding (×10^9^/L)	
≤10	18 (18.6 %)
11–20	35 (36.1 %)
21–50	37 (38.1 %)
51–100	7 (7.2 %)
Urea level at the time of bleeding (mmol/L)	13.7 (9.1–19.8)
Prothrombin time at the time of bleeding (%)	56 (43.5–70.2)
Concurrent anticoagulant and/or antiplatelet treatment	8 (8.2 %)
Preventive anticoagulant treatment	5 (5.1 %)
Curative anticoagulant treatment	2 (2.1 %)
Antiplatelet agent	2 (2.1 %)
Activated protein C	1 (1 %)

Variables are expressed as median (interquartile range) or number (percentage)

*ICU* intensive care unit, *WHO* World Health Organization

**Table 4:** Comparisons between patients with and without ICU-acquired severe bleeding (*n* = 279, after excluding 17 patients with bleeding on admission)

Variable	ICU-acquired severe bleeding *n* = 40 patients	No ICU-acquired severe bleeding *n* = 239 patients	*p*
Age	66.5 (57.2–73)	62 (54–69)	0.61
Male gender (%)	27 (67.5 %)	156 (65.3 %)	0.86
Underlying malignancy			1
Hematologic malignancy	33 (82.5 %)	196 (82 %)	
Solid tumor	7 (17.5 %)	43 (18 %)	
Bone marrow transplantation	5 (12.5 %)	34 (14.2 %)	0.87
Reasons for ICU admission			
Sepsis	15 (37.5 %)	124 (51.9 %)	0.12
Others	25 (62.5 %)	115 (48.1 %)	0.12
Anticoagulant and/or antiplatelet treatment	8 (20 %)	57 (23.8 %)	0.69
Admission severity scores			
SAPS II (points)	69 (34.2–80)	51 (37–57)	0.57
SOFA (points)	8 (6–11)	7 (5–10.7)	0.32
Modified SOFA (points)	7 (3–8)	4 (1–7)	0.31
Admission biological values			
Platelet count (×10^9^/L)	50 (26–84)	26 (13–53)	<0.001
Leukopenia	31 (77.5 %)	133 (55.6 %)	0.009
Bilirubin (μmol/L)	21 (11–77.7)	19 (11–40.7)	0.36
Serum urea (mmol/L)	10.9 (7.4–16.5)	9.7 (6.8–18.6)	0.20
Prothrombin time (%)	62 (44–75)	65 (50.2–77)	0.41
Organ failures during ICU stay			
Nadir platelet count (×10^9^/L)^a^	15 (7–27)	10 (6–19)	0.014
Organ failure supports			
Renal replacement therapy	17 (42.5 %)	100 (42 %)	1
Non-invasive ventilation	15 (37.5 %)	102 (42.7 %)	0.60
Invasive ventilation	28 (70 %)	139 (58.2 %)	0.16
Vasopressors	30 (75 %)	152 (63.6 %)	0.21
In-ICU mortality	20 (50 %)	86 (36 %)	0.11

Variables are expressed as median (interquartile range) or number (percentage). The modified SOFA score did not include the platelet component

*ICU* intensive care unit, *SAPS II* simplified acute physiology score II, *SOFA* sequential organ failure assessment score

^a^The nadir platelet count was the lowest platelet count recorded prior to the first severe bleeding event, or during the whole ICU stay for patients without severe bleeding

## Discussion

A number of studies have underscored the high prevalence and the poor prognostic value of thrombocytopenia in the ICU [1–3, 5, 6, 11–15]. Beyond absolute thrombocytopenia, decline in platelet count over the first days in the ICU, the so-called relative thrombocytopenia, also represents a reliable prognostic factor in addition to single measurements [2, 3, 11]. This suggests that the prognostic value of thrombocytopenia might not only be fully related to its depth, but also to the underlying pathophysiological process leading to platelet consumption or destruction [16]. It is noteworthy that all studies about thrombocytopenia in the ICU and the eventual indication of platelet transfusions have been performed in general cohorts of patients for whom decrease in platelet counts was mainly related to peripheral mechanisms [2–5, 17]. To the best of our knowledge, this is the first study that specifically addresses the transfusion management in cancer patients with hypoproliferative thrombocytopenia in the ICU.

The risk of bleeding events in the setting of thrombocytopenia underlies the use of prophylactic platelet transfusions. The current guidelines about the management of platelet transfusions in hypoproliferative thrombocytopenia are mainly derived from studies in patients with hematological malignancies requiring chemotherapy or undergoing allogeneic or autologous hematopoietic stem cell transplantation [18, 19]. Studies in hematology have been able to correlate the nadir platelet count with bleeding, the risk of severe hemorrhage clearly becoming much higher when platelet count drops below 10 × 10^9^/L [20–22]. In addition, various associated conditions are likely to increase the risk not only of bleeding in oncologic and hematologic thrombocytopenic patients, including the duration of thrombocytopenia, but also altered functional status, bone marrow metastasis, allogeneic bone marrow transplantation, a recent history of bleeding, hypoalbuminemia, and treatment with drugs affecting platelet function [21, 23]. Over the last two decades, several studies have addressed the indications of prophylactic platelet transfusion in clinically stable patients without active bleeding. Compared to the once traditional trigger of 20 × 10^9^/L, these studies have proven the safety and the cost-effectiveness of a more restrictive trigger of 10 × 10^9^/L in patients with prolonged thrombocytopenia related to acute myeloid leukemia [24–27]. An exclusive therapeutic policy was recently proposed to restrict platelet transfusion to documented bleeding [22, 28]. However, such a restrictive transfusion policy was associated with an increased risk of moderate-to-severe bleeding in patients with prolonged and profound thrombocytopenia as seen in acute leukemia.

The French Intensive Care Society recently provided expert recommendations in the management of platelet transfusions in the ICU [6]. Prophylactic platelet transfusion was recommended for central thrombocytopenia when platelet count fell below 20 × 10^9^/L. Accordingly, the large majority (80 %) of prophylactic platelet transfusions in the present study were performed at platelet counts thresholds lower than 20 × 10^9^/L. However, this recommendation was derived from studies performed in hematologic patients in stable conditions, and whether it can be safely implemented in critically ill cancer patients had not been addressed previously. Indeed, the quantitative relation between platelet count and hemorrhage remains unclear in critically ill patients in contrast to thrombocytopenic patients in stable conditions. Observational studies retrieved an increased risk of bleeding for severe thrombocytopenia (platelet count <50 × 10^9^/L), whereas the risk associated with moderate (50–100 × 10^9^/L) and mild (100–150 × 10^9^/L) thrombocytopenia was inconsistent [2, 4, 5, 13, 29]. Whether thrombocytopenia was directly involved in bleeding through the nadir platelet count or indirectly as a marker of severity remains unclear. Altogether, these results and ours suggest that ICU-acquired severe bleeding is a multifactorial process that is not only dependent on a sole platelet count. Most importantly, some additional factors such as uremia, other coagulation disorders, or anticoagulant treatments are frequently associated with the development of bleeding in the ICU, and should certainly be taken into account in the decision-making process of platelet transfusion in thrombocytopenic patients [12, 21].

A major obstacle to broad platelet transfusion lies in the limited availability of platelet concentrates. In addition, platelet transfusion may not be innocuous in the critically ill as supply of exogenous platelets may sustain or even exacerbate some pathophysiological thrombotic processes [15, 30–32]. Therefore, prophylactic platelet transfusion is usually not recommended in patients with exclusive peripheral thrombocytopenia [6]. As mentioned above, it is likely that both central and peripheral mechanisms are involved in thrombocytopenia of cancer patients, who commonly exhibit risk factors for platelet transfusion refractoriness such as spleen enlargement, bleeding, fever, infection, disseminated intravascular coagulation as well as the number of previous transfusions [33]. The low platelet recovery we observed in all three indications of transfusions probably resulted from fast consumption or destruction of exogenous platelets.

This study has several limitations. It was performed in a single tertiary care center with a particular case-mix of patients, and the period of inclusion was 7 years. However, our transfusion practices, based on previous studies in hematology and confirmed by the release of expert recommendations for critically ill patients, remained similar over the study period. Despite the fact that we primarily selected patients on the occurrence of platelet transfusions, this represents our common attitude in profound hypoproliferative thrombocytopenia and this probably allowed an exhaustive identification of patients. The major limit of the study obviously lies in its retrospective design, the results being highly dependent on the quality of records in the medical charts. However, we believe that automated extraction and quantitative variables from computerized charts as well as manual inquiry for other qualitative variable allowed us to perform a reliable collection of data. In contrast, duration, depth, and management of thrombocytopenia prior to ICU admission could not be accurately collected. The retrospective design may also have prevented a completely reproductive assessment of platelet recovery following transfusion. Indeed, we chose to collect the last platelet count prior to transfusion, and the next morning’s resulting platelet count. So the time interval between both platelet counts, as well as the timing of the transfusion in between, is likely to impact the difference. In the same way, this questions the exhaustiveness of collection of bleeding events. Undoubtedly, grade 3 and 4 bleeding events were significant enough to be reported in the charts, but grade 1 and 2 events might have been inconsistently mentioned. Finally, although our results suggest that the depth of thrombocytopenia was not an accurate predictor of severe bleeding, the retrospective design of the study and the quite low number of patients who bled precluded developing a more comprehensive and reliable multivariate predictive model of bleeding.

## Conclusion

In accordance with recent expert recommendations, most prophylactic platelet transfusions were given using restrictive thresholds of 10–20 × 10^9^/L in critically ill cancer patients with hypoproliferative thrombocytopenia. As a matter of fact, the risk of ICU-acquired severe bleeding appears hardly predictable with the depth of thrombocytopenia. Further prospective interventional studies are needed in order to reliably identify safe thresholds for platelet transfusion in critically patients with hypoproliferative thrombocytopenia in relation with the complex prediction of severe bleeding.
